# 

**Published:** 1880-07

**Authors:** 


### Table of Contents.

### ORIGINAL COMMUNICATIONS.

CARBOLIC ACID. By J. G. Westmoreland, M.D., Atlanta, Ga........... 193

### REPORTS OF SOCIETIES.

Cincinnati Academy of Medicine.................................................. 197
Baltimore Medical Association.................................................. 209
Medico-Chirurgical Society of Montreal.......................................... 213
The Rolla District Medical Society.............................................. 220

### SELECTIONS.

Clinical Lectures on Some Disorders Dependent upon Genital Irritation; Proper Treatment of Talipes, etc.................................. 223
Successful Amputation at the Hip-Joint for Sarcoma of the Thigh... 234
Antiseptic Surgery.................................................................. 238

### EDITORIAL.

Origin of Yellow Fever.............................................................. 242
Meteorological Report for June.................................................. 250

### BIBLIOGRAPHICAL

The Surgery, Surgical Pathology and Surgical Anatomy of the Female Pelvic Organs...................................................................... 251

### EXCERPTA.

Uræmia Treated by Pilocarpin.................................................. 251
Arsenic in Chorea.................................................................... 252
Benzoate of Soda in Parasitic Diseases ...................................... 253
Catarrh of the Bladder in Old People.......................................... 253
A New Treatment of Smallpox .................................................. 254
Hypodermic Quinia.................................................................. 255
Piscida Erythrina—Jamaica Dogwood.......................................... 255

